# Microbial and Heavy Metal Contaminant of Antidiabetic Herbal Preparations Formulated in Bangladesh

**DOI:** 10.1155/2015/243593

**Published:** 2015-10-26

**Authors:** Rausan Zamir, Anowar Hosen, M. Obayed Ullah, Nilufar Nahar

**Affiliations:** ^1^Department of Natural Sciences, Daffodil International University, Dhaka, Bangladesh; ^2^Department of Chemistry, University of Dhaka, Dhaka, Bangladesh; ^3^Department of Pharmacy, Daffodil International University, Dhaka, Bangladesh

## Abstract

The aim of the current study was to evaluate microbial contamination in terms of microbial load (total aerobic count and total coliform count) and specific pathogenic bacteria (*Salmonella* spp., *Escherichia coli*, *particularly Escherichia coli* 0157) in thirteen antidiabetic herbal preparations (ADHPs) from Dhaka City. All the thirteen ADHPs had been found contaminated with fungi and different pathogenic bacteria. From the data, it is found that only two of these preparations (ADHP-1 and ADHP-12) complied with the safety limit (as stated in different Pharmacopoeias and WHO guidelines) evaluated by all different microbial counts. None of these herbal preparations could assure the safety as all of them were contaminated by fungi. The overall safety regarding heavy metal content (Zn, Cu, Mn, Cr, Cd, and Pb) was assured as none of them exceeded the safety limit of the daily intake. Microbial contaminants in these herbal preparations pose a potential risk for human health and care should be taken in every step involved in the preparation of these herbal preparations to assure safety.

## 1. Introduction

Over the recent times, the popularity of herbal medicine is increased to such an extent that around 20% of world population is now using herbal medicine in different forms for different purposes [1]. In developing countries, it is estimated that 70–80% of the population somehow relay on nonconventional medicines mainly of herbal origins for their primary health care [2], as they are cheap and easily accessible [3]. Herbal preparations are produced from any raw or processed part of a plant, which includes leaves, stems, flowers, roots, and seeds, and in most of the cases it is a complex mixture of organic chemicals from natural sources [1, 4, 5]. As different plant parts are used in a herbal preparation, it may carry a large number of various kinds of microbes originating from soil usually adhering to different parts of herbs [5] (Table 1). Moreover, in some of the herbal preparations, particularly Ayurvedic formulation, the use of heavy metals is intentional, as some of these heavy metals are believed to have beneficial effect on our body. In general, most common contaminants are heavy metals, pesticides, microbes, and mycotoxins [6, 7].

The range of the usage [8] of herbal preparations is vast as they are frequently used in the treatment of several chronic diseases including type 2 diabetes (diabetes mellitus). Diabetes is a noncommunicable heterogeneous group of disorder and affects approximately 200 million individuals globally. Moreover, it is predicted that over 300 million people will be diabetic by 2015 [8, 9]. In general, this poses challenges to the health care and social welfare but, in particular, it is a huge challenge to a developing country like Bangladesh because of its limited resources and weak economy. The trend of uses of antidiabetic herbal preparations (mostly based on Ayurvedic and Unani formulary) is increasing day by day among the population of Bangladesh. In parallel, there is a rising concern regarding the safety and efficacy of these herbal preparations as most of them contain different contaminants including microbial contaminants and heavy metals (particularly in Ayurvedic preparation). In most of the developed countries, herbal preparations are defined as dietary supplement. As a result, unlike pharmaceutical preparations, manufacturers are producing, selling, and marketing herbal preparations without any evidence based scientific study regarding their safety and efficacy [1]. Although in several countries herbal medicine (a part of complementary and alternative medicine) is the officially approved system, there is no guidelines and regulations for assuring the safety of these preparations. The safety of these herbal preparations is very important because Ayurvedic formulation contains several heavy metals as therapeutic ingredients. But the use of these heavy metals beyond the limit could be toxic. Moreover, the level of microbial contamination of herbal preparations is dependent on the quality of the raw materials used and the manufacturing environment. Most raw materials for herbal preparations support some form of microbial growth, as medicinal plants used in herbal preparations provide nutrition to microorganisms and facilitate the multiplication of microorganism. In addition, inappropriate cleaning, unsuitable transportation, prolonged drying and storage, inadequate hygiene of producers, and congenital climatic conditions render the medicinal plants vulnerable to infestations and exposed them to many microbial contaminants. Inadvertent contamination, like fungal contamination during the production stage can also lead to deterioration in safety and quality as the risk of mycotoxin production, especially aflatoxin, may arise which has proven mutagenic, carcinogenic, teratogenic, neurotoxic, nephrotoxic, and immunosuppressive activities [10–16]. Therefore, it is important to evaluate the safety of these antidiabetic herbal preparations based on relevant scientific investigation. This research project focused on the safety of antidiabetic herbal preparations available in Bangladesh particularly related to heavy metal and microbial contamination.

In Bangladesh, several antidiabetic herbal preparations are readily available and are being used, but studies regarding microbial contaminants and heavy metal content in locally produced herbal preparations are limited. Therefore, it is necessary to evaluate microbial contamination as well as heavy metal content in some locally produced and widely used herbal preparations. In this study, we investigated the level of microbial contamination and heavy metal content present in antidiabetic herbal preparations widely used and formulated in Bangladesh.

## 2. Materials and Methods

### 2.1. Study Area and Sample Collection

Samples of thirteen antidiabetic herbal preparations (ADHPs) as finished commercial pack were purchased randomly from different herbal medicine outlets of Dhaka City. Initially, all the samples were prepared for analysis in the research laboratory, Daffodil International University (DIU), Dhaka, Bangladesh. Microbiological contamination and heavy metal content were analyzed in the Center for Advanced Research in Sciences (CARS), University of Dhaka, Bangladesh.

### 2.2. Determination of pH

The pH of different herbal preparations was determined by using microprocessor pH meter (HI 2210; Hanna Instrument, USA) as described earlier [17]. For pH determination, sample solution was prepared by dissolving 12.5 g in 100 mL sterile distilled water with shaking to obtain a homogenous solution. The pH of the solution of different herbal preparations was measured by microprocessor pH meter and the data are presented as the average of triplicate.

### 2.3. Preparation of Media

All the media for microbiological analysis were prepared according to the manufacture's guidelines and sterilized in an autoclave (CL-32S; ALP Co. Ltd., Japan) at 121°C for 40 minutes. The sterile media were dispensed or poured into sterilized Petri dishes or test tube as required. The sterility of the prepared media was confirmed by incubating blindly selected plates at 37°C for overnight.

### 2.4. Total Aerobic Bacterial Count and Total Coliform Count

Total aerobic bacterial count was performed to assess the quality and shelf life of the herbal formulation. Twenty-five (25) g of each sample was homogenized in 225 mL of sterile saline water. After that, 0.1 mL from twofold diluted samples was spread on a Petri dish containing Tryptic Soy Agar (TSA) (Oxoid Ltd., Hampshire, England) and incubated at 35°C for 24 hours for total aerobic bacterial count [18]. To assess the hygiene of the formulations, total coliform count was performed by spreading 0.1 mL of the sample (as used for total aerobic count) on MacConkey agar (Oxoid Ltd., Hampshire, England) and was incubated at 35°C and 42°C for 24 hours [19].

### 2.5. *Escherichia coli* 0157

Twenty-five (25) g of each sample was homogenized in 225 mL EC medium and incubated at 42°C for 20 hours. The enriched cultures were streaked onto Sorbitol MacConkey agar complemented with Cefixime and potassium tellurite supplement and characteristic colonies were subjected to biochemical tests (IMViC). Biochemically confirmed isolates were screened through caprylate-thallous agar (CTA) and CHROMagar. The colonies, which gave characteristic color, were subsequently serotyped by 0157 antisera.

### 2.6. *Escherichia coli* (Total)

Twenty-five (25) g of each sample was homogenized in 225 mL* Enterobacteria* enrichment broth-Mossel preenrichment medium and incubated at 35°C for 20 hours. One milliliter aliquots of preenriched cultures was mixed with nine milliliters of 2x EC medium and incubated at 35°C for 20 hours. One loop full of the culture was inoculated into 10 milliliters 1x EC medium with Durham fermentation tubes and incubated at 42°C for 20 hours. To isolate* E. coli*, one loop full of gas produced 1x EC culture broth was streaked on Chromocult agar (CTA) plates and developed typical colonies. The same preenrichment culture was used for isolation and characterization of coliform bacteria on Sorbitol MacConkey (SMAC) agar.

### 2.7. *Salmonella* spp

Twenty-five (25) g of each sample was homogenized in 225 mL of buffered peptone water and incubated at 35°C for 20 hours. One milliliter preenrichment culture was mixed with nine milliliters of Hanja Tetrathionate Broth and incubated at 35°C for 20 hours and nine milliliters of Rappaport-Vassiliadis Broth and incubated at 42°C for 20 hours. The culture broths were subsequently streaked onto Bismuth Sulfite Agar (BSA). For the isolation of each microorganism, original solution and 10^−2^ (hundred times diluted solution) were used for microbial limit test and pH of the samples was controlled within the range of 6.9–7.9 by adding NaOH or HCL.

### 2.8. Qualitative Fungi Counts

Fungi were identified on potato dextrose agar (PDA) (Oxoid Ltd., Hampshire, England) after incubation at 30°C for 5 days. Procedure and dilution were followed as described for total bacterial aerobic count. At the end of 5-day incubation, the fungal growth was observed under microscope [20].

### 2.9. Sample Preparation and Heavy Metal Analysis

Heavy metals were analyzed in flame atomizer based atomic absorption spectrometer using hollow cathode lamp as a radiation source. Accurately, 25 g of herbal preparation was transferred into silica crucible and kept in a muffle furnace for ashing at 700°C for 1 hour. The sample was then cooled down to room temperature and the heating process was repeated for three times. The ash was then dissolved by adding 5–10 mL of concentrated HCl and finally diluted the sample by 0.1 N HNO_3_ up to 25 mL. Finally, the sample was prepared for heavy metal analysis by filtering through Whatman filter paper.

For heavy metal analysis, the samples were aspirated through nebulizer and the absorbance was measured against a blank as a reference. Specific hollow cathode lamps were used to analyze Copper (wavelength 324.8 nm), Cadmium (wavelength 228.8 nm), Chromium (wavelength 357.9 nm), Manganese (wavelength 297.5 nm), Lead (wavelength 283.3 nm), and Zinc (wavelength 213.9 nm). Before analysis, the samples were diluted to the appropriate factor according to the detection limit of the Atomic Absorption Spectrophotometer (AAnalyst 200; Perkin Elmer, USA). Calibration curve was obtained using referent standard and all the measurements were run in triplicate for the samples and standards solutions.

## 3. Results and Discussion

### 3.1. Microbial Contamination

**Figure 1:**
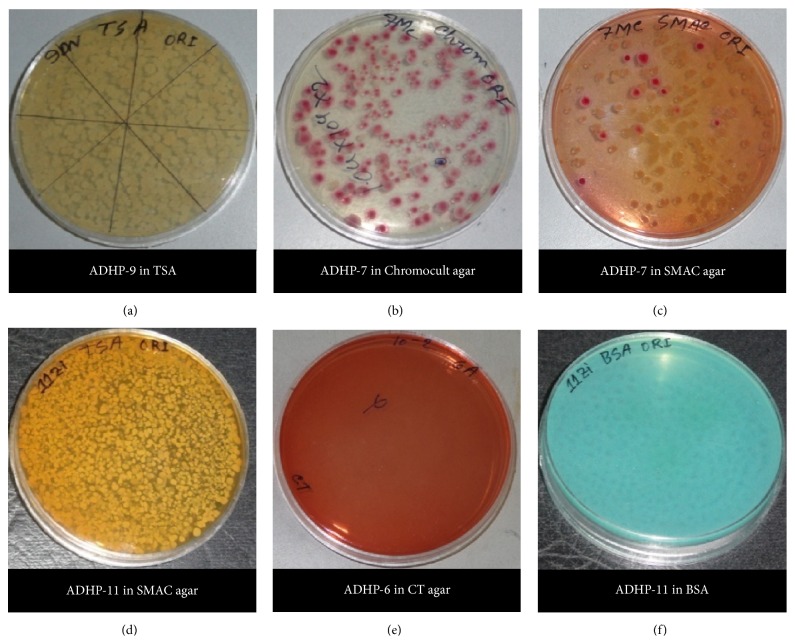
Incubation of antidiabetic herbal preparations (ADHPs) in different agar media for microbial count. (a) ADHP-9 incubated in Tryptic Soya Agar (TSA) plate for total aerobic count, (b) ADHP-7 incubated in Chromocult agar plate for total coliform count, (c) ADHP-7 and ADHP-11 incubated in Sorbitol MacConkey (SMAC) agar plate for total* E. coli* count, (e) ADHP-6 incubated in caprylate-thallous Agar (CTA) plate for* E. coli* 0157 count, and (f) ADHP-11 incubated in Bismuth Sulfite Agar (BSA) plate for* Salmonella* spp. count.

**Figure 2:**
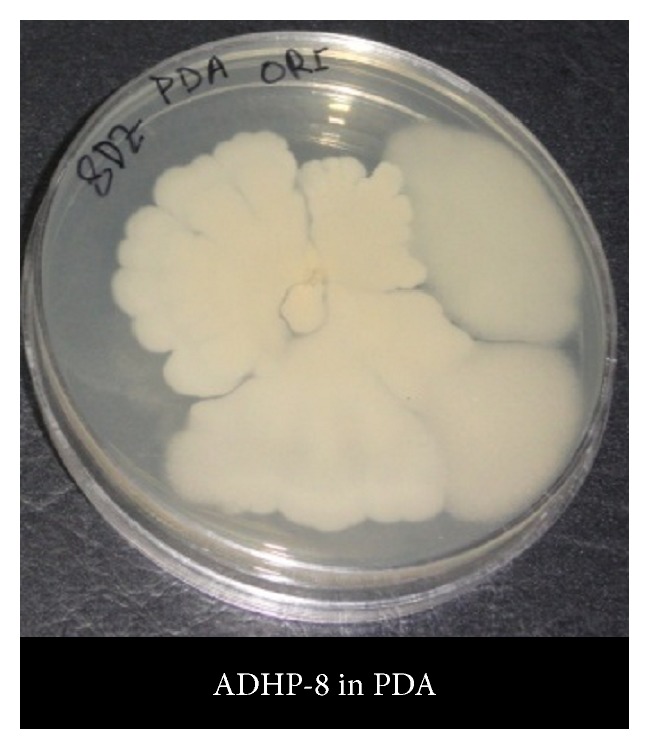
Incubation of ADHP-8 in potato dextrose agar (PDA).

For the evaluation of microbial contamination, total bacterial aerobic, total coliform, total* E. coli*,* E. coli* 0157, and* Salmonella* spp. count were determined (Figure 1). All the preparations showed different levels of total aerobic bacterial count and exceeded the safety limit according to USP (United States Pharmacopoeia) (Tables 2 and 3), but six of them (ADHP-2, ADHP-4, ADHP-6, ADHP-10, and ADHP-11) exceeded the safety limit as indicated by EP (European Pharmacopoeia) and WHO (World Health Organization) guidelines (Table 3) whereas two of them (ADHP-5 and ADHP-9) were in marginal level. Total coliform count is the indicator of faecal contamination and is found in six of the samples where they exceeded the safety limit (Tables 2 and 3). Total* E. coli* count, a specific Gram negative bacterial species count included in the range of total coliform count, also exceeded the safety limit in fifty percent of the studied preparation. Specific species counts such as* E. coli* 0157 and* Salmonella* spp. were found to be present in around 25% of the preparation (*E. coli* 0157 in ADHP-9, ADHP-11, and ADHP-13 and* Salmonella* spp. in ADHP-4) (Tables 2 and 3). Almost seventy percent of the total preparation studied (nine preparations) failed to comply with the safety limit at least in one method of microbial contamination evaluation like total microbial counts or specific species count. In this study, we counted microorganism in five different ways (total aerobic bacterial count, total coliform count, total* E. coli* count, specific* E. coli* 0157 count, and* Salmonella* spp. count), where ADHP-4 and ADHP-11 exceeded safety limit in four different microbial counting methods. ADHP-9 is in second position in failing the safety limit as it exceeded the safety limit evaluated by three different counts. At least in two different microbial counts, the level of microbial contamination was higher than the safety limit in ADHP-7, ADHP-10, and ADHP-13 as mentioned in the EP, USP, and WHO guidelines (Tables 2 and 3). From the data, it is found that two of these preparations (ADHP-1 and ADHP-12) only could be able to comply with the safety limit evaluated by all the different microbial counts. If we consider the presence of fungi in the preparation then none of these herbal preparations could comply with different standardizing body for the assurance of safety as all of the thirteen ADHPs have shown positive response in potato dextrose agar (PDA) (Figure 2).

The presence of large numbers of pathogenic bacteria in the studied herbal preparations indicates several windows to consider as a source of contamination. It is worth mentioning that the pH of all the preparations was within the suitable range (pH 5–8.5) which may appreciate bacterial growth [21]. The contamination could start at the initial phase of raw materials collection as soil influences bacterial growth in several ways. This initial contamination could be carried along to harvesting, drying, and storage. Moreover, during the preparation of finished preparation, the source of contamination includes personnel, equipments, and materials. Therefore, the process of raw material collections and processing of the raw materials and the process of manufacturing for finished preparation should ensure the highest possible level of hygiene to maintain the lowest possible level of pathogenic organism in the preparation and thereby assure the quality and safety of herbal preparation.

The level of microbial contamination is mentioned in different standards for publication including EP, USP, and WHO guideline to maintain the safety of herbal preparations (Table 3). Gram negative bacteria such as* Salmonella*,* Shigella,* and* E. coli* should be absent in the preparation. Moreover, the limit for coliforms is also mentioned, as it is the most reliable indicator of faecal contamination, which may indicate the possible presence of other harmful disease-causing organisms. The presence of fungi in herbal preparations under certain conditions may lead to the secretion of toxic metabolites such as mycotoxins, which when ingested, inhaled, or absorbed through the skin cause illness or human and animal death [22]. These mycotoxins possess substantial risk of carcinogenic, neurotoxic, immunotoxic, and mutagenic effects [11–16]. It is reported that a substantial amount of medicinal plants is contaminated naturally by fungi from soil and environment and thereby may contain mycotoxins [10]. As most of the herbal preparation majorly contains medicinal plants, it is important to assure that the levels of mycotoxins are below the safety limit as set by different bodies. For conclusive remark, we further need to determine the level of mycotoxins in these herbal preparations.

### 3.2. Heavy Metal Content

In this study, we determined heavy metal (Cu, Cd, Cr, Mn, Pb, and Zn) contents in different ADHPs to identify any potential risk of the accumulation of these heavy metals leading to toxicity (Table 4). All the thirteen ADHPs contain Copper (Cu), Chromium (Cr), Manganese (Mn), Lead (Pb), and Zinc (Zn) in some levels with few exceptions (Cr in ADHP-9 and Zn in ADHP-7 and ADHP-12 were below detection level) (Table 4). The amount of cadmium was below detection level in all the preparations except ADHP-4 and ADHP-13. There are several regulatory bodies that set specific allowable limit for heavy metal content in herbal and tradition preparations based on different guidelines and this permissible limit varies among these regulatory bodies (Tables 5 and 7). It is found that lead content in almost all of the samples (except ADHP-7 and ADHP-9) exceeded the permissible limit if we consider the stringiest limit of Chines Pharmacopoeia (Tables 4 and 5). Even if we consider a more relax permissible limit for lead (WHO and US FDA guidelines; Table 5), one-third of the total ADHPs (ADHP-4, ADHP-6, ADHP-8, and ADHP-13) failed to comply with the safety limit. Lead, a highly toxic environmental pollutant, can affect the function of various biomolecules by forming complex with them. Moreover, excess lead exposure may be responsible for poor muscle coordination, gastrointestinal symptoms, brain and kidneys damage, hearing and vision impairments, and reproductive defects [23–25]. Cadmium content was below detection level in all of the ADHP samples other than ADHP-4 and ADHP-13. Unfortunately, these two (ADHP-4 and ADHP-13) samples also failed to comply with safety based on cadmium content (Tables 4 and 5). Cadmium toxicity could induces tissue injury [26–28], epigenetic changes in DNA expression [29–31], hypertension [32], diabetes [33], apoptosis [34], and insulin resistance [35, 36]. Moreover, excess cadmium may inhibit or upregulate transport pathways [37–39] and heme synthesis [40]. According to JECFA (The Joint FAO/WHO Expert Committee on Food Additives) heavy metal limits for herbal dietary supplements, none of these formulations contains heavy metals in such a level, which could exceed the daily allowable intake (Tables 6 and 7). Considering all of these guidelines, it turned out that only two ADHP samples (ADHP-7 and ADHP-9) contain heavy metals in safe level. Metals are natural components of soils and some of them (Cu, Mn, and Zn) are necessary for micronutrients of plant growth while others (Cd, Cr, and Pb) are not but could be accumulated in plants at toxic level [41–43]. As the major components of these herbal preparations are plants, the presence of heavy metals in ADHPs is very relevant. Some of the identified metals (Zn, Cu, Mn, and Cr) have important biological role in the body.

## 4. Conclusion

Based on at least two of the evaluation experiments, all of the ADHPs were found to be contaminated with microorganism and/or fungi, which pose potential threat to human health. The heavy metal content particularly Lead in ADHP samples was alarming as almost all of them failed to comply with safety limit. Further detection of other heavy metal content like arsenic and mercury could give us a broader understanding of heavy metal contamination in these herbal preparations. In general, this contamination may come from raw materials, during processing of raw materials and manufacturing of finished products due to the production environment. In a nutshell, finished products reach consumers with zero contamination; quality has to be maintained throughout the process beginning from the selection of raw material to the final product. Taking these facts into consideration, regulatory agencies should come forward and take the necessary measures to ensure the safety of finished herbal preparations.

## Figures and Tables

**Table 1:** Composition of different antidiabetic herbal preparations (ADHPs) and daily adult dose as indicated on the label of the products.

Sample ID	Brand name	Composition of the preparation as indicated on the label of finished product	Daily adult (70 kg bw) dose in g	pH
ADHP-1	Diacare	*Bambusa bambos* (surface of inner skin) *Gymnema sylvestre *(leaf) *Acacia arabica *(leaf) *Rumex vesicarius* (seeds)	1.5	5.86

ADHP-2	Ziabit	*Natrum sulfuricum* (a constitutional homeopathy remedy) Glauber's salts (sodium sulphate decahydrate)	1	5.36

ADHP-3	Insucontrol	*Syzygium cumini* (seeds) Ferrous sulphate (salt) *Rumex vesicarius* (seeds)	3	5.44

ADHP-4	Dolabi	*Gymnema sylvestre* (leaf) *Bambusa bambos* (surface of inner skin) *Rumex vesicarius* (seeds) Asphalt	2.5	6.74

ADHP-5	Diazym	*Gymnema sylvester* (leaf) Asphalt *Mytilus margaritiferus*	2.5	6.25

ADHP-6	Alisa	*Allium sativum* *Allium cepa* *Mangifera indica* (leaf) *Myristica fragrans* (dried kernel of the seed) *Syzygium aromaticum* (flower)	3.75	8.18

ADHP-7	DaruchiniJamseed	*Cinnamomum zeylanicum* (bark) *Accacia acuminate* (seeds)	4.25	5.81

ADHP-8	Garlic	*Allium sativum* (bulbs) *Allium cepa* (bulbs) *Syzygium cumini* (seed) *Mangifera indica* (leaf) *Myristica fragrans* (dried kernel of the seed) *Syzygium aromaticum* (flower)	3.5	7.83

ADHP-9	Methicrash	*Trigonella foenum-graecum* (seed)	30	6.70

ADHP-10	Diano	*Bambusa arundinacea* (surface of inner skin) *Rumex vesicarius* (seed) *Gymnema sylvestre* (leaf) Hen's egg shell Ferrous sulphate *Mytilus margaritiferus* Asphalt	2.5	7.10

ADHP-11	Azardiracha Indica	*Azadirachta indica* (leaf extract)	2.5	6.35

ADHP-12	Cuzium Jam	*Cinnamomum zeylanicum* (bark) *Accacia acuminate* (seeds)	4	5.72

ADHP-13	Silaraj	*Salvia haematodes* (bark and root) Asphalt Calcined iron oxide Calcined stannum	2.5	7.56

**Table 2:** Microbial assessment of different ADHPs.

Sample name	Total aerobic bacterial count/mL	Total coliform count/mL	Total *E. coli* count/mL	*E. coli* 0157 count/mL	*Salmonella* spp. count/mL
ADHP-1	2.0 × 10^4^	2.0 × 10^2^	Negative	Negative	Negative
ADHP-2	1.4 × 10^6^	Negative	Negative	Negative	Negative
ADHP-3	4.0 × 10^4^	2.7 × 10^3^, 1.7 × 10^3^ (w, c)	Negative	Negative	Negative
ADHP-4	5.72 × 10^6^	8.4 × 10^3^ (P) 8.00 × 10^2^ (W)	3.1 × 10^3^ (W) 6.0 × 10^2^ (P)	Negative	2.75 × 10^3^
ADHP-5	2.9 × 10^5^	Negative	Negative	Negative	Negative
ADHP-6	5.7 × 10^5^	Negative	Negative	Negative	Negative
ADHP-7	7.08 × 10^4^	2.08 × 10^4^	2.34 × 10^4^	Negative	Negative
ADHP-8	5.36 × 10^4^	Negative	Negative	Negative	Negative
ADHP-9	2.5 × 10^5^	2.89 × 10^3^	9.5 × 10^2^	4.4 × 10^2^	Negative
ADHP-10	8.3 × 10^5^	8.0 × 10^2^	2.9 × 10^2^	Negative	Negative
ADHP-11	1.38 × 10^6^	3.93 × 10^4^	1.34 × 10^4^ (P) 7.8 × 10^3^ (W)	2.1 × 10^3^ (P)	Negative
ADHP-12	2.03 × 10^4^	Negative	Negative	Negative	Negative
ADHP-13	1.87 × 10^6^	5.0 × 10^3^	5.0 × 10^2^ (P) 1.6 × 10^3^ (W)	1 × 10^2^ (P)	Negative

**Table 3:** Microbial limits for finished herbal/botanical preparations (in colony-forming units/gram (cfu/g) or colony-forming units/mL (cfu/mL)) (current as of July 2014).

Reference	EP category C	USP	WHO
Product	Product with Ingredients demonstrated to fail Catergory B w/Processing/Pretreatment	Containing botanical ingredients	Herbal materials for internal use

Total aerobic microbial count	10^5^ (maximum acceptance limit: 5 × 10^5^)	10^4^	10^5^
Total combined yeast and mold count	10^4^ (maximum acceptance limit: 5 × 10^4^)	10^3^	10^3^
Enterobacterial count (bile-tolerant Gram negative bacteria)	10^4^	NA	10^3^ (other than *E. coli*)
*Escherichia coli*	Absence in 1 g	Absence in 10 g	10 in 1 g
*Salmonella* spp.	Absence in 25 g	Absence in 10 g	Absence in 1 g
*Staphylococcus aureus*	NA	NA	NA
*Clostridia*	NA	NA	Absence in 1 g
Shigella	NA	NA	Absence in 1 g

EP: European Pharmacopoeia Edition 8.0, 5.1.8 (microbiological quality of herbal medicinal products for oral use and extracts used in their preparation), 2013.

USP: United States Pharmacopeial Convention, USP-NF 37-32, 2014.

WHO: World Health Organization, WHO Guidelines for Assessing Quality of Herbal Medicines with Reference to Contaminants and Residues, 2007.

NA: not assigned.

**Table 4:** Heavy metal content of investigated ADHP samples.

Sample ID	Zn (ppm)	Cu (ppm)	Mn (ppm)	Cr (ppm)	Cd (ppm)	Pb (ppm)
ADHP-1	5.38	5.38	6.5	9.25	BDL	8.50^¶^
ADHP-2	3.75	10.00	9.25	4.63	BDL	8.50^¶^
ADHP-3	2.00	10.50	6.63	4.38	BDL	7.00^¶^
ADHP-4	12.50	3.50	7.75	28.25	2.75^*∗*§¶^	41.38^*∗*^
ADHP-5	2.88	4.88	1.63	8.25	BDL	6.63^¶^
ADHP-6	3.75	4.50	8.50	4.00	BDL	13.38^*∗*¶^
ADHP-7	BDL	4.13	3.00	2.88	BDL	3.75
ADHP-8	2.75	3.75	0.88	6.00	BDL	11.50^*∗*¶^
ADHP-9	2.88	8.88	6.88	BDL	BDL	3.88
ADHP-10	2	7.25	8.50	11.75	BDL	9.88^¶^
ADHP-11	2.38	4.50	8.00	2.13	BDL	5.75^¶^
ADHP-12	BDL	4.25	4.63	2.50	BDL	9.38^¶^
ADHP-13	10.50	3.13	6.00	24.63	1.38^*∗*§¶^	33.50^*∗*§¶^

BDL: below detection level; ^*∗*^exceed WHO and US FDA permission limit; ^§^exceed HAS Singapore permission limit; ^¶^ exceed Chines Pharmacopoeia permission limit.

**Table 5:** Permissible limit of heavy metal in herbal drugs.

Heavy/toxic metal	WHO	US FDA	HSA Singapore	Chinese Pharmacopoeia
Cadmium	0.20 ppm	0.30 ppm	0.05 ppm	0.30 ppm
Lead	10.00 ppm	10.00 ppm	20.00 ppm	5.00 ppm
Arsenic	10.00 ppm	10.00 ppm	5.00 ppm	2.00 ppm
Mercury	1.00 ppm	1.0 ppm	0.50 ppm	0.20 ppm
Copper	20.00 ppm	20.00 ppm	150.00 ppm	20.00 ppm
Zinc	50.00 ppm	50.00 ppm	—	—

US FDA: United States Food and Drug Administration; HAS: Health Science Authority.

**Table 6:** Heavy metal content of investigated ADHP samples and the daily safe intake of different heavy metals.

Sample ID	Cumulative daily adult dose of preparation^*∗*^ (g)	Daily adult intake of heavy metal (in *μ*g) as calculated form the dose indicated on the label of the finished product					
		Zn	Cu	Mn	Cr	Cd	Pb
ADHP-1	1.50	8.06	8.06	9.75	13.88	—	12.75
ADHP-2	1.00	3.75	10.00	9.25	4.63	—	8.50
ADHP-3	3.00	6.00	31.50	19.88	13.13	—	21.00
ADHP-4	2.50	31.25	8.75	19.38	70.63	6.88	103.44
ADHP-5	2.50	7.19	12.19	4.06	20.63	—	16.56
ADHP-6	3.75	14.06	16.88	31.88	15.00	—	50.16
ADHP-7	4.00	—	16.50	12.00	11.00	—	15.00
ADHP-8	3.50	9.63	13.13	3.06	21.00	—	40.25
ADHP-9	30.00	86.25	266.25	206.25	—	—	116.25
ADHP-10	2.50	5.00	18.13	21.25	29.38	—	24.69
ADHP-11	2.50	5.94	11.25	20.00	5.31	—	14.38
ADHP-12	4.00	—	17.00	18.50	10.00	—	37.50
ADHP-13	2.50	26.25	7.81	15.00	61.56	3.44	83.75

^*∗*^This dose is calculated as indicated on the label of the finished product; BDL: below detection level.

**Table 7:** JECFA heavy metal limits for herbal dietary supplements.

Heavy metals	Stated limit (PTWI, weekly)	Calculated daily limit (adult, 70 kg)
Arsenic	15 *μ*g inorganic arsenic/kg bw	150 *μ*g
Cadmium	7 *μ*g cadmium/kg bw	70 *μ*g
Lead	25 *μ*g lead/kg bw	250 *μ*g
Mercury	1.6 *μ*g methylmercury/kg bw	16 *μ*g

JECFA: The Joint FAO/WHO Expert Committee on Food Additives; PTWI: provisional tolerable weekly intake.
